# A Perspective on Reagent Diversity and Non-covalent Binding of Reactive Carbonyl Species (RCS) and Effector Reagents in Non-enzymatic Glycation (NEG): Mechanistic Considerations and Implications for Future Research

**DOI:** 10.3389/fchem.2017.00039

**Published:** 2017-06-30

**Authors:** Kenneth J. Rodnick, R. W. Holman, Pamela S. Ropski, Mingdong Huang, Arthur L. M. Swislocki

**Affiliations:** ^1^Department of Biological Sciences, Idaho State UniversityPocatello, ID, United States; ^2^Department of Chemistry, Idaho State UniversityPocatello, ID, United States; ^3^College of Chemistry, Fuzhou UniversityFujian, China; ^4^Division of Endocrinology and Metabolism, Department of Internal Medicine, School of Medicine, University of California, DavisDavis, CA, United States; ^5^Veterans Affairs Northern California Health Care SystemMartinez, CA, United States

**Keywords:** reactive carbonyl species, glycation, diabetes, glucose, phosphate, mechanism, glycation gap

## Abstract

This perspective focuses on illustrating the underappreciated connections between reactive carbonyl species (RCS), initial binding in the nonenzymatic glycation (NEG) process, and nonenzymatic covalent protein modification (here termed NECPM). While glucose is the central species involved in NEG, recent studies indicate that the initially-bound glucose species in the NEG of human hemoglobin (HbA) and human serum albumin (HSA) are non-RCS ring-closed isomers. The ring-opened glucose, an RCS structure that reacts in the NEG process, is most likely generated from previously-bound ring-closed isomers undergoing concerted acid/base reactions while bound to protein. The generation of the glucose RCS can involve concomitantly-bound physiological species (e.g., inorganic phosphate, water, etc.); here termed effector reagents. Extant NEG schemes do not account for these recent findings. In addition, effector reagent reactions with glucose in the serum and erythrocyte cytosol can generate RCS (e.g., glyoxal, glyceraldehyde, etc.). Recent research has shown that these RCS covalently modify proteins *in vivo* via NECPM mechanisms. A general scheme that reflects both the reagent and mechanistic diversity that can lead to NEG and NECPM is presented here. A perspective that accounts for the relationships between RCS, NEG, and NECPM can facilitate the understanding of site selectivity, may help explain overall glycation rates, and may have implications for the clinical assessment/control of diabetes mellitus. In view of this perspective, concentrations of ribose, fructose, Pi, bicarbonate, counter ions, and the resulting RCS generated within intracellular and extracellular compartments may be of importance and of clinical relevance. Future research is also proposed.

## Introduction

Reactive carbonyl species (RCS) are electrophiles containing one or more carbonyl functional groups, typically aldehydes and/or ketones, which are present under physiological conditions. RCS exist *in vivo* (a) as enzymatic and/or metabolic products (Tessier, 2010; Turk, 2010); (b) from exogenous sources (Uribarri et al., 2007); and (c) from nonenzymatic glycation processes (Tessier, 2010). Certain RCS exist as species in equilibrium with other non-RCS isomers and, as such, can be termed transient RCS. The ring-opened isomer of the monosaccharide D-glucose is a transient RCS because it equilibrates with other less reactive ring-closed isomers that do not contain a reactive carbonyl. A biological system that exhibits negative implications as a result of high RCS concentration is said to be under “carbonyl stress” (Suzuki and Miyata, 1999; Turk, 2010). The biochemical implications of carbonyl stress include, but are not limited to: (a) increased oxidation of carbohydrates and lipids (oxidative stress); (b) increases in steady-state level of reactive species, including reactive oxidative species (ROS), advanced lipoxidation end products (ALE), and advanced glycation end products (AGE); along with (c) perturbed cellular metabolism (Yim et al., 2001; Thornalley, 2005; Shumaev et al., 2009). Carbonyl stress has clinical implications in many chronic and degenerative diseases including diabetes, obesity, kidney and heart diseases, atherosclerosis, and neurodegenerative diseases.

The transient ring-opened glucose RCS isomer reacts non-enzymatically with intracellular and extracellular proteins to form glycated proteins, both *in vivo* (Bookchin and Gallop, 1968; Bunn et al., 1975) and *in vitro* (Baynes et al., 1989) over prolonged periods of time (weeks to months) via a multi-reaction process termed non-enzymatic glycation (NEG). For this perspective, we define the NEG process as the four stage process that involves the initial binding of glucose, followed by Schiff base formation, then Amadori formation, and finally the formation of AGE. One result of the process is the production of covalently-modified protein(s), which can alter protein function (Philippe and Bourdon, 2011) and is thought to be linked to the chronic complications of diabetes mellitus (Forbes and Cooper, 2013). The measurement of glycated human hemoglobin (i.e., HbA1_C_) is currently a cornerstone of the management of diabetes mellitus. It is used for diagnostic and management purposes (Swislocki, 2012). The pathophysiological implications of NEG also include age-related chronic diseases such as microangiopathy, retinopathy, and nephropathy (Brownlee, 1995; Baynes, 2001).

Glucose is not, however, the only species that can non-covalently bind proteins that can lead to covalently-modified protein. When other sugars (e.g., ribose, fructose, G6P, etc.) and non-sugar metabolites (e.g., glyceraldehyde, glyoxal, etc.) bind to protein and ultimately generate covalently-modified proteins, we refer to the process as non-enzymatic covalent protein modification (NECPM). The NECPM process, as defined here, differs from NEG in that NEG involves only glucose whereas NECPM involves non-glucose species. In NECPM, the bound species may, or may not, follow the four-stage NEG process, may have a different degree of reversibility relative to NEG, and may lead to a different array of AGE relative to NEG. The reason for the NEG/NECPM distinction is to emphasize that different reagents via different mechanistic pathways can lead to different covalently-modified proteins. For example, most of the non-glucose RCS metabolites are aldehydes that cannot cyclize but have the potential for significant hydrate formation (Swenson and Barker, 1971; Kitayama et al., 2002). In addition, the binding and follow-up chemistry of hydrates is completely unrelated to the NEG process. The NEG versus NECPM distinction is also of value because of the clinical significance of glucose as the predominant glycating agent *in vivo* and the realization that sites for protein modification are not identical for glucose versus glyceraldehyde. As an example, Lys82 on the β-chain of HbA is not glycated by glucose but is covalently modified by glyceraldehyde (Acharya and Manning, 1980; Delpierre et al., 2004; Ito et al., 2010). We posit that glucose follows an NEG pathway whereas glyceraldehyde follows a disparate NECPM pathway.

The goal of this perspective is to illustrate the diversity of reagents and the multiplicity of mechanisms that can be involved in early processes that lead to the covalent modification of proteins. For this purpose, HbA and human serum albumin (HSA) serve as model proteins. These reagents include glucose (in NEG), non-glucose sugars, RCS formed *in vivo* (and participate in NECPM), and effector reagents. We define an effector reagent as a small molecule that concomitantly binds with a reagent in a protein pocket and can facilitate or inhibit covalent bond making or bond breaking (e.g., water, Pi, bicarbonate, etc.). As an interdisciplinary team of authors, we posit concise, comprehensive schemes for NEG and NECPM that reflect recent research. Finally, we put forward future research directions and issues to be considered.

## Initial glucose/protein binding in NEG: the role of RCS

Glucose undergoes reversible mutarotation in aqueous media, whereby five different isomers interconvert, including a pair of ring-closed pyranoses (α and β) and a pair of ring-closed furanoses (α and β). The ring-closed species are not RCS and are not sufficiently electrophilic to significantly react with protein amino acid residues. The fifth structure, through which the four ring-closed isomers interconvert, is a ring-opened isomer containing a free aldehyde group. This species is a transient RCS that is sufficiently electrophilic to react with amino acid residues in NEG.

**Figure 1:**
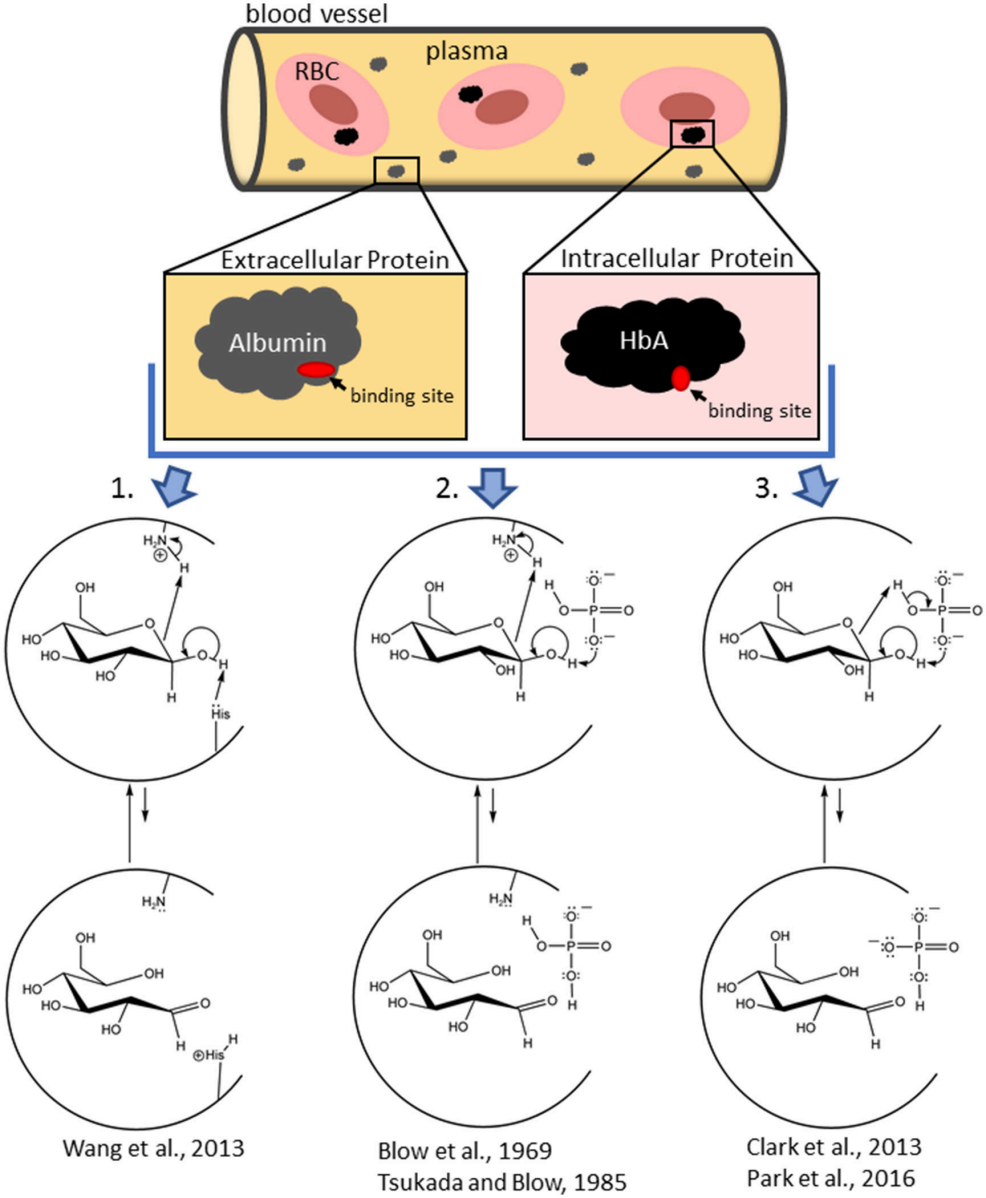
Schemes showing the mechanistic diversity for the production of the non-covalently bound reactive carbonyl species (RCS) in a protein pocket (either at the surface of the protein or in an internal pocket) from initially bound glucopyranose in intracellular (hemoglobin) and extracellular (albumin) proteins. In an amino-acid-residue-mediated mechanism (1), the protein (shown as a semi-circle) itself is acting as both the acid and base. In the Pi-mediated mechanism (2), an amino acid residue acts as the acid while the concomitantly bound Pi (as an effector reagent) acts as the base. In another Pi-mediated mechanism (3), Pi bridges the bound glucopyranose and acts as the effector reagent for both the acid and base chemistry. Water or bicarbonate, etc. can also play the role of the effector reagent (in fact, water is the effector reagent in glucose mutarotation in aqueous solution, Silva et al., 2006). Each of these mechanisms are examples of concerted reactions that do not generate net charge and is similar to a charge relay enzymatic mechanism, such as that for chymotrypsin (Blow et al., 1969; Tsukuda and Blow, 1985; Park et al., 2016). Mechanisms are depicted as taking place with β-glucopyranose, but comparable mechanisms with α-glucopyranose also occur.

Results from HSA (Wang et al., 2013) illustrate that the initially-bound glucose species are the non-RCS ring-closed isomers that then ring open once bound. This observation was confirmed by Clark et al. (2013) for glucose interactions with HbA and is consistent with studies of glucose and other monosaccharides binding to several enzymes (D-xylose isomerase, Lee et al., 1990; Allen et al., 1994; galactose mutarotase, Thoden et al., 1994; and phosphoglucoseisomerase, Lee and Jeffrey, 2005). In fact, we are unaware of any detailed mechanistic investigation in support of the direct binding of the transient, acyclic glucose isomer to protein. The direct binding (and subsequent reaction) of the ring-opened isomer of glucose in NEG is very unlikely because the equilibrium concentration of glucose in the ring-opened form in aqueous solution is 0.002% (Hayward and Angyal, 1977; Bunn and Higgins, 1981), corresponding to a concentration of just 0.12 μM in human plasma/serum. This concentration is likely an overestimate of the actual concentration of available ring-opened isomer because the ring-opened isomer rapidly re-ring closes. The lifetime of the ring-opened isomer is exceedingly short as it reverts (via intramolecular processes) to ring-closed isomers at a rate faster than the ^1^HNMR time scale at 300 MHz (75 ms, or ~10^−4^ s, Bryant, 1983). Moreover, re-ring closure is likely faster than the process of binding and then reacting once bound. In contrast, the ring-closed isomers of glucose are at 50,000 times the concentration, are long-lived, and bind better than does the ring-opened isomer (Clark et al., 2013). Thus, the ring-opened RCS structure is most likely generated from previously-bound, ring-closed isomers (Clark et al., 2013; Wang et al., 2013; e.g., Figure 1). Once generated, the transient RCS must then react with protein before it either reverses to a non-electrophilic, ring-closed isomer or exits the protein pocket. Extant schemes for NEG depict the ring-opened isomer as the singular species that directly binds to protein, when, in fact, overwhelmingly it is ring-closed, non-RCS isomers that bind and then are reversibly converted to the ring-opened glucose isomer while bound (Clark et al., 2013; Wang et al., 2013). These findings are not reflected in historic or even in recent NEG schemes (Rodwell et al., 2015; Welch et al., 2016).

## Glucose RCS generation while bound to protein

### How does a glucopyranose ring open while bound?

When pure α-glucopyranose is placed in DMSO (a polar aprotic solvent), mutarotation does not proceed (Ballash and Robertson, 1973). Thus, spontaneous ring opening does not occur. Therefore, generation of the reactive ring open RCS of glucose, whether in aqueous media or while bound to a protein requires an effector reagent. Water, as an effector reagent, is thought to bridge the anomeric OH and the hemiacetal oxygen of a glucopyranose and serve as both an acid and a base in a concerted process (Silva et al., 2006; Qian, 2012). A typical protein pocket has a dielectric constant of a 2–4 and, as such, there are often no water molecules in the pocket (Simonson and Brooks, 1995). That said, one or more water molecules can exist within protein pockets from incomplete desolvation of the protein upon glucose binding (Mowbray and Cole, 1992). Further, water complexes of Pi and/or glucose, and/or the concomitant binding of independent water molecules are also possible (Silva et al., 2006; Qian, 2012). For surface proteins with water exposure, water should be readily available. Thus, water can serve as an effector reagent to assist ring opening of glucose. However, effector reagents other than water (serving as an acid, base, or acid/base catalyzing species) should also be considered. For example, effector reagents for HbA include physiological anion/buffers such as Pi (Figure 1), bicarbonate, 2,3-bisphosphoglycerate (2,3-BPG), etc. The ring opening of a bound glucopyranose requires the deprotonation of the OH on the anomeric carbon and the protonation of the hemi-acetal oxygen (on C5). Ideally, this occurs without the formation of new ionic intermediates in a low dielectric environment. Therefore, irrespective of the identity of the effector reagent, concerted reactions are most likely.

Given the presence of acidic and basic amino acid residues in protein pockets, effector reagents are not necessary. For example, the amine forms (R-NH_2_) of internal lysine and histidine side chains, the N-terminal amino acid residue, can act as bases, while ammonium ions (R-${\text{NH}}_{3}^{+}$) of internal lysines, N-terminal amino acids and/or Pi can act as acids (Figure 1). That said, the common external effector reagents (water, Pi, etc.) have greater geometric freedom to accommodate the proper alignment for concerted ring opening and also have greater buffering ability (Figure 1). Thus, rates of NEG and NECPM should be faster as effector reagent concentration increases.

In fact, Pi is known to accelerate HbA glycation, though the mechanism(s) are uncertain (Gil et al., 1988, 2004; Kunika et al., 1989). In recent work (Clark et al., 2013; Smith et al., in preparation), ^31^P and ^1^HNMR of model reactions and computational assessment of reaction thermodynamics and protein/substrate interactions highlight that: (1) Pi and a glucopyranose can undergo reversible, concomitant binding to HbA glycation sites, generating an array of HbA-bound Pi/glucopyranose complexes of comparable energy with different geometries, (2) the Pi within the HbA-bound Pi/glucopyranose complex achieves a geometry to ring-open the bound glucopyranose, and (3) one such geometry has Pi bridge a protein-bound glucopyranose, enabling Pi to abstract the proton on the anomeric OH while protonating the hemi-acetal oxygen in a concerted process (Figure 1).

We assert that the observed site selectivity for glycation with glucose (NEG) may be related to the ability to generate the necessary electrophile (via ring-opening of the glucopyranose while bound), which varies from site-to-site.

## The potential of non-glucose RCS in NECPM

Glucose is not the only monosaccharide that is a physiological, transient RCS. Fructose (Bunn and Higgins, 1981; Wang et al., 2013), ribose (Bunn and Higgins, 1981), and G6P (glucose-6-phosphate, Haney and Bunn, 1976) may also undergo ring opening while bound and potentially proceed toward covalent protein modification. Bunn et al. (1975) and Swamy et al. (1993) show that each of these species covalently modify proteins (HbA and lens crystalline, respectively) more extensively than glucose on a per molecule basis. It is reasonable to assert that ring-closed non-RCS isomers of these monosaccharides are the predominate species that initially bind. As such, they likely ring open while bound to generate the bound RCS needed for further reaction (Wang et al., 2013). Moreover, when glyceraldehyde is used instead of glucose for *in vitro* protein modification, covalent modification is much more extensive than that for glucose (Hamada et al., 1996). This is likely because glyceraldehyde precedes via an NECPM mechanism that may involve the binding of both aldehydes and their hydrates which proceed through later mechanisms unrelated to those in NEG.

The formation of RCS derived from monosaccharide degradation *prior to protein interaction* needs to be considered. At pH = 7, Pi enhances the conversion of α-glucopyranose to β-glucopyranose in D_2_O by a factor of 21 (Bailey et al., 1970). Once Pi generates a ring-opened glucose isomer (before binding to protein), a second Pi can further react with the acyclic glucose isomer to form additional RCS (Thornalley et al., 1999; Henning et al., 2014). When summing the degradation products from the above work and extrapolating to include fructose, ribose, and G6P degradation products, more than 20 Pi-promoted RCS can theoretically be formed prior to protein binding. Binding of such RCS is consistent with the rapid modification of protein by species like glyceraldehyde (Acharya and Manning, 1980). Further support comes from preliminary computations in our laboratory that at least a subset of these RCS undergo binding to HbA and HSA (with similar exothermicity to glucopyranose binding) with geometries suitable to react such that the RCS can act as electrophiles and covalently modify HbA at Val1 of the β-chain of HbA–the site specific for HbA1c formation by glucose (Bookchin and Gallop, 1968; Bunn et al., 1975). Various researchers (Toi et al., 1967; Oya et al., 1999; Thornalley, 2005; Nasiri et al., 2011) have highlighted that certain of these RCS, (e.g., glyoxal and methylglyoxal) react with arginine residues and lead to the covalent modification of proteins (which we, here, would assert are NECPM processes). This highlights yet another distinction between NEG and NECPM, as glucose (in NEG) does not react readily with arginine.

Arguments for the importance of these RCS and NECPM are: (1) degradation products from glucose, fructose, and ribose are far more electrophilic than glucose (e.g., methylglyoxal is 20,000 more reactive than glucose, Thornalley et al., 1999); (2) unlike the glucopyranoses, many of the RCS do not need to be modified, while bound, to make an electrophile and therefore, there is no need for the concomitant binding of an effector reagent; and (3) the residence time demand (the required amount of time bound in order to react; Tummino and Copeland, 2008) within a protein pocket is much shorter for most of these RCS than for glucose. On the other hand, the expected concentrations of these RCS in plasma will likely be in the low μM range (Henning et al., 2014), well below the normal physiological concentrations of glucose (4–5 mM). However, while each independent RCS (other than glucose) has a comparatively low concentration, it is the sum of *all* monosaccharide degradation products whose concentration matters most (10–20 μM). Intracellular concentrations of these RCS are unknown.

## Nucleophile formation in NEG

The bound, electrophilic RCS may react with a nucleophilic N-terminal amino acid residue or an R-NH_2_ amino acid side chain of a lysine or possibly an arginine in NEG and NECPM. At physiological pH, these amino acid residues are often in their non-nucleophilic, ammonium ion form (R-${\text{NH}}_{3}^{+}$). This important detail is often missing from current NEG schemes and is not consistent with basic nucleophile requirements and progression in NEG to the Schiff base. Physiological anions (such as Pi and bicarbonate) can produce nucleophiles by deprotonating ammonium ions, a role not previously highlighted.

## Perspective summary and considerations

### Perspective on reagent and mechanistic diversity

**Figure 2:**
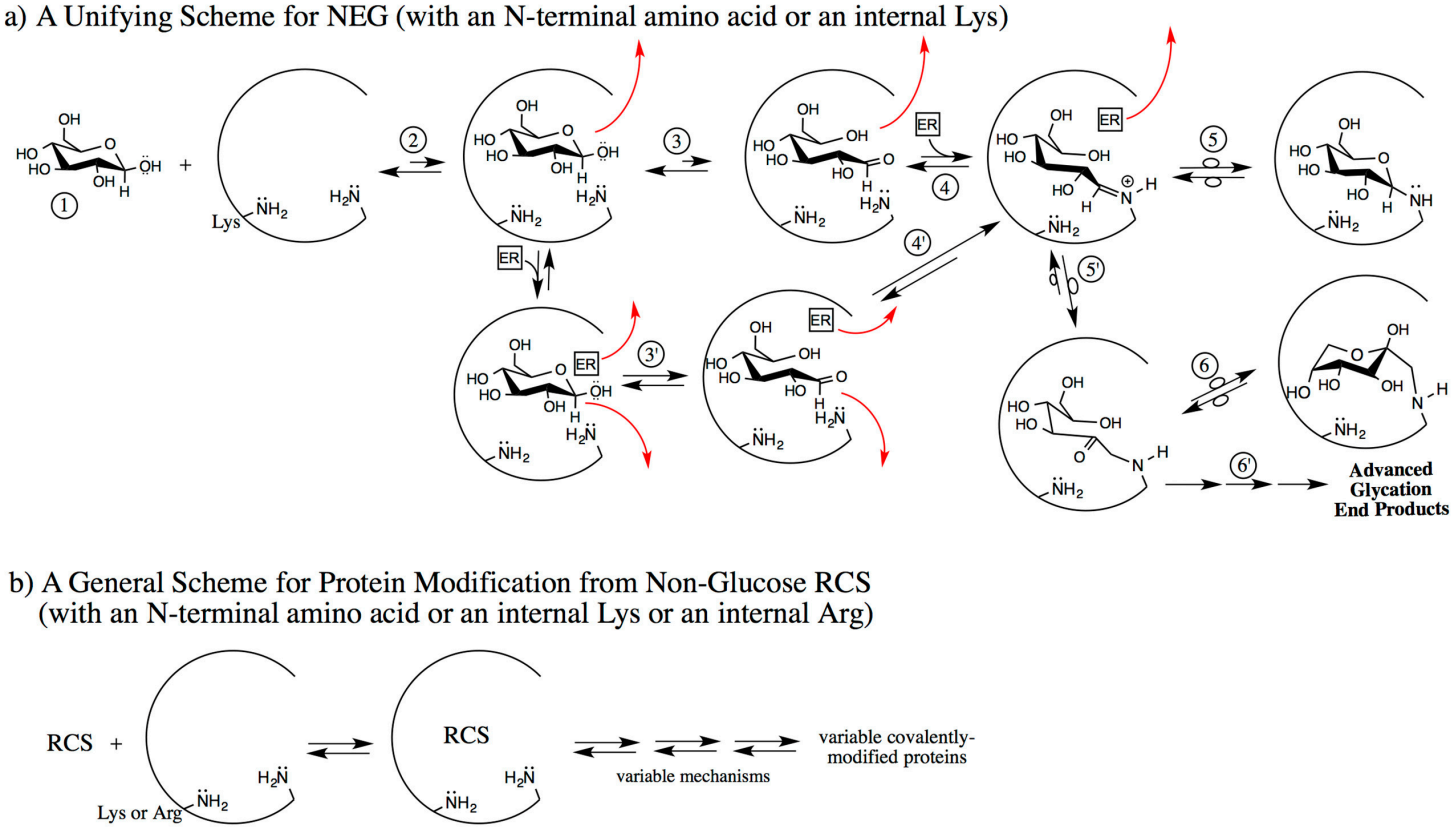
Two proposed schemes for nonenzymatic covalent protein modification emphasizing the early, non-covalent interactions. **(a)** A non-RCS precursor (1) is exposed to a protein pocket (an internal pocket or at the surface of the protein); here the example is β-glucopyranose (but it can be fructose, ribose, G6P, etc.). The non-RCS precursor noncovalently binds (2). It may dissociate from the protein pocket or it may proceed via 3 or 3′ [if an effector reagent (ER) such as Pi, water or bicarbonate, etc. were to concomitantly bind]. Under the influence of amino acid residues only, acid/base reactions with the initially-bound non-RCS precursor generate a noncovalently bound, transient, reactive RCS (3). In this case, the ring-opened glucose isomer may dissociate from the protein pocket or it may proceed via 4 to the Schiff base. An effector reagent such as Pi (mono or dibasic), water, bicarbonate, 2,3-BPG or other physiological species can concomitantly bind with the non-RCS precursor and then function as an acid/base reagent to generate a non-covalently bound, transient, reactive RCS; in this case, the ring-opened glucose isomer (3′). Any of the bound reagents may dissociate at any point in the 3 or 3′ transition to 5. If the ring-opened glucose does not dissociate, it can proceed to the Schiff base via 4′. The bound, transient, reactive RCS (4, 4′), in this case, the ring-opened glucose isomer, can form either without an ER (2–3) or by an ER (2–3′). Schiff base can form with facilitation if an ER concomitantly binds at this point (4) or via 4′ where the ER did not dissociate. The covalently bound, protonated Schiff base has three fates: it can reverse back to noncovalently bound species (4), it can isomerize to a cyclic glycosylamine via 5, or it can undergo Amadori formation via 5′. Note: Amadori formation may or may not involve an ER. The Amadori intermediate has three fates; it can reverse back to the Schiff base (5′), it can isomerize to a cyclic Amadori (6), or it can go on via multiple mechanisms to AGE (6′). **(b)** Non-glucose RCS and/or hydrates of the RCS are exposed to a protein pocket, can noncovalently bind, and then proceed by variable mechanisms (which may or may not include ER) to generate variable types of covalently-modified proteins. A subset of the RCS are sufficiently electrophilic to enable arginine residues to be covalently modified. These processes do not necessarily go through Schiff bases and/or Amadori intermediates. The covalent modification may or may not be reversible and may or may not involve AGE-type structures.

Many RCS besides the ring-opened glucose isomer and an array of different effector reagents can potentially be involved. From a mechanistic perspective, bases may include Pi, histidine, lysine/N-terminal amino acid amines, and water, while reagents acting as acids may include water, Pi, bicarbonate, 2,3 BPG, and potentially others. The potential nucleophiles are the amines of N-terminal amino acid residues and internal lysines and arginines (if reacted with aldehydic RCS). Therefore, reactions to generate bound RCS can involve many combinations of reagents, via an array of different mechanisms. We propose that non-covalent binding mechanisms are likely to differ from protein-to-protein and from site-to-site on the same protein under the same conditions. This is significant because with multiple mechanisms there is potential for multiple rate-determining steps. A new scheme is proposed (Figure 2) for NEG and NECPM processes to account for this reagent and mechanistic diversity.

### Perspective on NEG site selectivity

What dictates the site selectivity of covalent protein modification and the extent of NEG? What creates a glycation hot spot [(i.e., the N-terminal valine on the β-chain of HbA forming HbA1c (Delpierre et al., 2004) and the Lys195 on human albumin Wang et al., 2013]? The site-to-site binding affinity of the transient RCS electrophile cannot explain site selectivity (Clark et al., 2013). We propose that both the binding affinity for RCS and effector reagents and their binding geometries within protein pockets differ from site-to-site. Moreover, the identity of the effector reagent and the intrinsic mechanism may vary from site-to-site.

### Perspective on NEG rate

For transient RCS, the likelihood of arriving at the proper geometry with the proper amino acid charge state and a suitably proximate nucleophile within the lifetime of the bound species (e.g., before re-ring-closure or dissociation of glucose from the protein pocket) is low. These contingencies might help explain why the NEG process is so slow.

### Perspective on clinical implications

The term “glycation gap” refers to the difference between measured HbA1c and HbA1c predicted from a concurrent measure of NEG of serum proteins (i.e., fructosamine), or other indices of glycemic control. We posit that differential concentrations of effector reagents in erythrocytes vs. serum (e.g., Pi and 2,3-BPG) for certain patients may be relevant to understanding the mechanistic basis for the glycation gap. A further consideration is the differential concentrations of Na^+^, Ca^2+^, and Mg^2+^ (serum vs. intracellular) that can affect Pi availability as an effector reagent (Walser, 1961).

### Perspective on future directions: what needs to be considered?

1. Because the generation of RCS while bound to protein has a temporal component, the measurement of time-dependent geometry/energetics and residence time of bound reacting species utilizing Molecular Dynamics simulations (Dror et al., 2012) and/or 2D NMR methods (Williamson, 2013) is advised.
2. To date, the only variables assumed to determine the degree of intracellular protein (i.e., HbA) modification are glucose concentration, protein lifetime, and cell permeability (Cohen et al., 2008; Malka et al., 2016). In view of this perspective, concentrations of ribose, fructose, Pi, bicarbonate, other cations and anions, and the resulting RCS generated within intracellular and extracellular compartments may be of importance and of clinical relevance. Clinical measures of the aforementioned species vs. disease progression in diabetic patients and a comparison with normal controls would be insightful.
3. If RCS generated from reactions of monosaccharides are important, then reagents that selectively remove RCS from either serum or the erythrocyte cytosol may decrease the extent of protein modification (specifically by NECPM). The development of potential RCS scavengers (carbonyl trapping compounds), AGE formation inhibitors, and chemicals capable of breaking AGE-protein crosslinks is ongoing (Cho et al., 2007), and together with mechanistic investigations of various effector reagents under physiological conditions, may provide novel strategies to manage health complications related to excessive NEG and NECPM.
4. Research on effector reagent implications on equilibria between reacting bound species (Schiff base, protonated Schiff base, cyclic glycosylamines, etc.) and the resulting impact on site selectivity at stages after initial binding is warranted (especially as extended to proteins beyond HbA and HSA, and also to enzymes).

In summary, this perspective illustrates far greater reagent diversity and mechanistic complexity than previously thought, even when considering only implications in the initial binding stages. This presents a persistent challenge to analyze, describe, understand and potentially reduce the extent of NEG or NECPM processes.

## Author contributions

KR, RH, and PR conceived the project and led the writing of the perspective with input and contributions from MH and AS.

### Conflict of interest statement

The authors declare that the research was conducted in the absence of any commercial or financial relationships that could be construed as a potential conflict of interest.

## References

[B1] Acharya A. E. Manning J. M. (1980). Reactivity of the amino groups of carbonmonoxyhemoglobin S with glyceraldehyde. J. Biol. Chem. 255, 1406–1412. 7354037

[B2] Allen K. N. Lavie A. Glasfeld A. Tanada T. N. Gerrity D. P. Carlson S. C.. (1994). Role of divalent metal ion in sugar binding, ring opening, and isomerization by D-xylose isomerase: replacement of a catalytic metal by an amino acid. Biochemistry 33, 1488–1494. 10.1021/bi00172a027 7906142

[B3] Bailey J. M. Fishman P. H. Pentchev P. G. (1970). Anomalous mutarotation of glucose 6-phosphate. An example of intramolecular catalysis. Biochemistry 9, 1189–1194. 10.1021/bi00807a020 4392154

[B4] Ballash N. M. Robertson E. B. (1973). The mutarotation of glucose in dimethylsulfoxide and water mixtures. Can. J. Chem. 51, 556–564. 10.1139/v73-085

[B5] Baynes J. W. (2001). The role of SGEs in aging: causation or correlation. Exp. Gerontol. 36, 1527–1537. 10.1016/S0531-5565(01)00138-3 11525875

[B6] Baynes J. W. Watkins N. G. Fisher C. I. Hull C. J. Patrick J. S. Ahmed M. U. (1989). The Amadori product on protein; structure and reactions. The maillard reaction in aging, diabetes, and nutrition. Prog. Clin. Biol. Res. 304, 43–67. 2675036

[B7] Blow D. M. Birktoft J. J. Hartley B. S. (1969). Role of a buried acid group in mechanism of action of chymotrypsin. Nature 221, 337–340. 10.1038/221337a0 5764436

[B8] Bookchin R. M. Gallop P. M. (1968). Structure of hemoglobin A1c – nature of the N-terminal beta chain blocking group. Biochem. Biophys. Res. Commun. 32, 86–93. 10.1016/0006-291X(68)90430-0 4874776

[B9] Brownlee M. (1995). Advanced protein glycation on diabetes and aging. Ann. Rev. Med. 46, 223–234. 10.1146/annurev.med.46.1.223 7598459

[B10] Bryant R. G. (1983). The NMR time scale. J. Chem. Educ. 60, 933–935. 10.1021/ed060p933

[B11] Bunn H. F. Haney D. N. Gabbay K. H. Gallop P. M. (1975). Further identification of the nature and linkage of the carbohydrate in hemoglobin A1c. Biochem. Biophys. Res. Commun. 67, 103–109. 10.1016/0006-291X(75)90289-2 1201013

[B12] Bunn H. F. Higgins P. J. (1981). Reaction of monosaccharides with proteins: Possible evolutionary significance. Science 213, 222–224. 10.1126/science.12192669 12192669

[B13] Cho S. J. Roman G. Yeboah F. Konishi Y. (2007). The road to advanced glycation endproducts: a mechanistic perspective. Curr. Med. Chem. 14, 1653–1671. 10.2174/092986707780830989 17584071

[B14] Clark S. L. D. Santin A. E. Bryant P. A. Holman R. W. Rodnick K. J. (2013). The initial non-covalent binding of glucose to human hemoglobin in nonenzymatic glycation. Glycobiology 23, 1250–1259. 10.1093/glycob/cwt061 23926230

[B15] Cohen R. M. Franco R. S. Khera P. K. Smith E. P. Lindsell C. J. Ciraolo P. J.. (2008). Red cell life span heterogeneity in hematologically normal people is sufficient to alter HbA1c. Blood 112, 4284–4291. 10.1182/blood-2008-04-154112 18694998 PMC2581997

[B16] Delpierre G. Vertommen D. Communi D. Rider M. H. Van Schaftingen E. (2004). Identification of fructosamine residues deglycated by fructosamine-3-kinase in human hemoglobin. J. Biol. Chem. 279, 27613–27620. 10.1074/jbc.M402091200 15102834

[B17] Dror R. O. Dirks R. M. Grossman J. P. Xu H. Shaw D. E. (2012). Biomolecular simulation: a computational microscope for molecular biology. Annu. Rev. Biophys. 41, 429–452. 10.1146/annurev-biophys-042910-155245 22577825

[B18] Forbes J. M. Cooper M. E. (2013). Mechanisms of diabetic complications. Physiol. Rev. 93, 137–188. 10.1152/physrev.00045.2011 23303908

[B19] Gil H. Mata-Segreda J. F. Schowen R. L. (1988). Proton transfer is not rate-limiting in buffer-induced non-enzymic glucation of hemoglobin. J. Am. Chem. Soc. 110, 8265–8266. 10.1021/ja00232a065

[B20] Gil H. Vásquez B. Peña M. Uzcategui J. (2004). Effect of buffer carbonate and arsenate on the kinetics of glycation of human hemoglobin. J. Phys. Organ. Chem. 17, 537–540. 10.1002/poc.772

[B21] Hamada Y. Araki N. Koh N. Nakamura J. Horiuchi S. Hotta N. (1996). Rapid formation of advanced glycation end products by intermediate metabolites if glycolytic pathway and polyol pathway. Biochem. Biophys. Res. Commun. 228, 539–543. 10.1006/bbrc.1996.1695 8920948

[B22] Haney D. N. Bunn H. F. (1976). Glycosylation of hemoglobin *in vitro*: affinity labeling of hemoglobin by glucose-6-phosphate. Proc. Natl. Acad. Sci. U.S.A. 73, 3534–3538. 10.1073/pnas.73.10.3534 1068465 PMC431151

[B23] Hayward L. D. Angyal S. J. (1977). A symmetry rule for the circular dichroism of reducing sugars, and the proportion of carbonyl forms in aqueous solutions thereof. Carbohydr. Res. 53, 13–20. 10.1016/S0008-6215(00)85450-6

[B24] Henning C. Liehr K. Ulrich C. Glomb M. A. (2014). Extending the spectrum of α-dicarboyl compounds *in vivo*. J. Biol. Chem. 289, 28676–26688. 10.1074/jbc.M114.563593 25164824 PMC4192516

[B25] Ito S. Nakahari T. Yamamoto D. (2010). Relationship between impaired glycation and the N-terminal structure of the Hb Görwihl [β5 (A2) Pro → Ala] variant. Hemoglobin 34, 151–156. 10.3109/03630261003676785 20353350

[B26] Kitayama T. Ushikubo T. Morihashi K. Kikuchi O. (2002). Conformation and parity-violating energy of hydrated D-glyceraldehyde in aqueous solution. J. Mol. Struct. 586, 1–7. 10.1016/S0166-1280(01)00803-X

[B27] Kunika K. Itakura M. Yamashita K. (1989). Inorgainic phosphate accelerates hemoglobin A1c synthesis. Life Sci. 45, 623–630. 10.1016/0024-3205(89)90048-9 2770417

[B28] Lee C. Bagdasarian M. Meng M. Zeikus J. G. (1990). Catalytic mechanism of xylose (glucose) isomerase from *Clostridium thermosulfurogenes*. Characterization of the structural gene and function of active site histidine. J. Biol. Chem. 265, 19082–19090. 2229064

[B29] Lee J. H. Jeffrey C. J. (2005). The crystal structure of rabbit phosphoglucose isomerase complexed with D-sorbitol-6-phosphate, an analog of the open chain form of D-glucose-6-phosphate. Protein Sci. 14, 727–734. 10.1110/ps.041070205 15689508 PMC2279277

[B30] Malka R. Nathan D. M. Higgins J. M. (2016). Mechanistic modeling of hemoglobin glycation and red cell kinetics enables personalized diabetes monitoring. Sci. Transl. Med. 8, 359ra130. 10.1126/scitranslmed.aaf9304 27708063 PMC5714656

[B31] Mowbray S. L. Cole L. B. (1992). 1.7 Å X-ray structure of the periplasmic ribose receptor from *Escherichia coli*. J. Mol. Biol. 225, 155–175. 10.1016/0022-2836(92)91033-L 1583688

[B32] Nasiri R. Field M. J. Zahedi M. Moosavi-Movahedi A. A. (2011). Cross-linking mechanism of arginine and lysine with α,β-dicarbonyl compounds in aqueous solution. J. Phys. Chem. 115, 13542–13555. 10.1021/jp205558d 21970517

[B33] Oya T. Hattori N. Mizuno Y. Miyata S. Maeda S. Osawa T. (1999). Methylglyoxal modification of protein. Chemical and immunological characterization of methylglyoxal-arginine adducts. J. Biol. Chem. 274, 18492–18502. 10.1074/jbc.274.26.18492 10373458

[B34] Park B. Holman R. W. Slade T. Murdock M. Rodnick K. J. Swislocki A. L. M. (2016). A biochemistry question-guided derivation of a potential mechanism for HbA1c formation in diabetes mellitus leading to a data-driven clinical diagnosis. J. Chem. Educ. 93, 795–797. 10.1021/acs.jchemed.5b00554

[B35] Philippe R. Bourdon E. (2011). The glycation of albumin: structural and functional impacts. Biochimie 93, 645–658. 10.1016/j.biochi.2010.12.003 21167901

[B36] Qian X. (2012). Mechanisms and energetics for Brønsted acid-catalyzed glucose condensation, dehydration and isomerization reactions. Top. Catal. 55, 218–226. 10.1007/s11244-012-9790-6

[B37] Rodwell V. Bender D. Botham K. M. Kennelly P. J. Weil P. A. (2015). Harpers Illustrated Biochemistry 30th Edition. Columbus, OH: McGraw-Hill Education.

[B38] Shumaev K. B. Gubkina S. A. Kumskova E. M. Shepelkova G. S. Ruuge E. K. Lankin V. Z. (2009). Superoxide formation as a result of interaction of L-lysine with dicarbonyl compounds and its possible mechanism. Biochemistry 74, 461–466. 10.1134/S0006297909040154 19463101

[B39] Silva A. M. da Silva E. C. da Silva C. O. (2006). A theoretical study of glucose mutarotation in aqueous solution. Carbohydr. Res. 341, 1029–1040. 10.1016/j.carres.2006.02.035 16584715

[B40] Simonson T. Brooks C. L. (1995). Charge screening and the dielectric constant of proteins: insights from molecular dynamics. J. Am. Chem. Soc. 118, 8452–8458. 10.1021/ja960884f

[B41] Suzuki D. Miyata T. (1999). Carbonyl stress in the pathogenesis of diabetic nephropathy. Int. Med. 38, 309–314. 10.2169/internalmedicine.38.309 10361902

[B42] Swamy M. S. Tsai C. Abraham A. Abraham E. C. (1993). Glycation mediated lens crystalline aggregation and cross-linking by various sugar and sugar phosphates *in vitro*. Exp. Eye Res. 56, 177–185. 10.1006/exer.1993.1025 8462651

[B43] Swenson C. A. Barker R. (1971). Proportion of keto and aldehyde forms in solutions of sugar and sugar phosphates. Biochemistry 10, 3151–3154. 10.1021/bi00792a026 5126930

[B44] Swislocki A. (2012). HbA1c and metabolic syndrome. Metab. Syndr. Relat. Disord. 10, 391–393. 10.1089/met.2012.1503 23025691

[B45] Tessier F. J. (2010). The Maillard reaction in the human body. the main discoveries and factors that affect glycation. Pathol. Biol. (Paris) 58, 214–219. 10.1016/j.patbio.2009.09.01 19896783

[B46] Thoden J. B. Timson D. J. Reece R. J. Holden H. M. (1994). Molecular structure of human galactose mutarotase. J. Biol. Chem. 279, 23431–23437. 10.1074/jbc.M402347200 15026423

[B47] Thornalley P. J. (2005). Dicarbonyl intermediates in the maillard reaction, Ann. N.Y. Acad. Sci. 1043, 111–117. 10.1196/annals.1333.014 16037229

[B48] Thornalley P. J. Langborg A. Minhas H. S. (1999). Formation of glyoxal, methylglyoxal and 3-deoxyglucosone in the glycation of proteins by glucose. Biochem. J. 344, 109–116. 10.1042/bj3440109 10548540 PMC1220620

[B49] Toi K. Bynum E. Norris E. Itano H. A. (1967). Studies on the chemical modification of arginine. the reaction of 1, 2-cyclohexanedione with arginine and arginyl residues of proteins. J. Biol. Chem. 242, 1036–1043. 6020430

[B50] Tsukuda H. Blow D. M. (1985). Structure of α-chymotrypsin refined at 1.68 Å resolution. J. Mol. Biol. 184, 703–711. 10.1016/0022-2836(85)90314-6 4046030

[B51] Tummino P. J. Copeland R. A. (2008). Residence time of receptor-ligand complexes and its effect on biological function. Biochemistry 47, 5481–5492. 10.1021/bi8002023 18412369

[B52] Turk Z. (2010). Glycotoxines, carbonyl stress and relevance to diabetes and its complications. Physiol. Res. 59, 147–156. 19537931 10.33549/physiolres.931585

[B53] Uribarri J. Cai W. Peppa M. Goodman S. Ferrucci L. Striker G.. (2007). Circulating glycotoxins and dietary advanced glycation endproducts: two links to inflammatory response, oxidative stress, and aging. J. Gerontol. A Biol. Sci. Med. Sci. 62, 427–433. 10.1093/gerona/62.4.427 17452738 PMC2645629

[B54] Walser M. (1961). Ion association. VI. Interactions between calcium, magnesium, inorganic phosphate, citrate, and protein in normal human plasma. J. Clin. Invest. 40, 723–730. 10.1172/JCI104306 13782899 PMC373170

[B55] Wang Y. Haiyang Y. Shi X. Luo Z. Lin D. Huang M. (2013). Structural mechanism of ring-opening reaction by glucose by human serum albumin. J. Biol. Chem. 288, 15980–15987. 10.1074/jbc.M113.467027 23592780 PMC3668753

[B56] Welch K. J. Kirkman M. S. Sacks D. B. (2016). Role of glycated proteins in the diagnosis and management of diabetes: Research gaps and future directions. Diabetes Care 39, 1299–1306. 10.2337/dc15-2727 27457632 PMC4955935

[B57] Williamson M. P. (2013). Using chemical shift perturbation to characterize ligand binding. Prog. Nucl. Magn. Reson. Spectrosc. 73, 1–16. 10.1016/j.pnmrs.2013.02.001 23962882

[B58] Yim M. B. Yim H. S. Lee C. Kang S. O. Chock P. B. (2001). Protein glycation: creation of catalytic sites for free radical generation. Ann. N.Y. Acad. Sci. 928, 48–53. 10.1111/j.1749-6632.2001.tb05634.x 11795527

